# Interleukine-17 Modulates Neurogenesis and Behavior Following Exposure to Trauma in Mice

**DOI:** 10.3390/cells11030343

**Published:** 2022-01-20

**Authors:** Yehoshua Willinger, Gadi Turgeman

**Affiliations:** 1 Department of Molecular Biology, Faculty of Natural Sciences, Ariel University, Ariel 40700, Israel; yehoshuawi@ariel.ac.il; 2 Medical School, Ariel University, Ariel 40700, Israel

**Keywords:** interleukin-17, neurogenesis, post-traumatic stress disorder, social behavior

## Abstract

Post-traumatic stress disorder (PTSD) is a psychiatric disorder accompanied by deficits in cognitive and social skills. Adult hippocampal neurogenesis is a lifelong phenomenon, with new neurons being formed in the granular cell layer of the dentate gyrus. Impaired neurogenesis is associated with multiple behavioral disorders including Alzheimer’s disease and schizophrenia. PTSD patients often present hippocampal atrophy and animal models clearly present impaired neurogenesis. Previous studies on PTSD patients demonstrated elevated levels of Th17 cells and plasma levels of the pro-inflammatory cytokine interleukin-17A (IL-17A). Since IL-17A can impair neurogenesis in mice, we thus hypothesized that decreasing the serum levels of IL-17A will increase hippocampal neurogenesis and alleviate symptoms in a murine model of PTSD. Surprisingly, our results showed that attempting to neutralize IL-17A with an antibody resulted in increased serum levels of IL-17A, while targeting IL-23, the upstream regulator of IL-17, did lower the levels of IL-17A in trauma-exposed mice. As expected, increased levels of serum IL-17A (in anti-IL-17A treated mice) resulted in impaired neurogenesis, reflected by reduced number of proliferating Ki67^+^ neural progenitors and newly formed DCX^+^ neurons, which was correlated with increased expression of *Hes1*. Nevertheless, increased maturation was noted by the expression of *Slit2* and *Ache*. In contrast, treatment with anti-IL-23 indeed resulted in increased neurogenesis. Behaviorally, both treatments did not affect trauma-related freezing behavior but did affect trauma-related social deficits. Unexpectedly, increased levels of serum IL-17A (in anti-IL-17A treated mice) prevented social deficits in trauma-exposed mice while anti-IL-23 exacerbated these deficits. We thus conclude that IL-17 is involved in regulating neurogenesis following exposure to stress but may be important in maintaining social behavior.

## 1. Introduction

Neural stem cells (NSC), characterized by their long-term self-renewal and neuronal differentiation potential, have been shown to persist throughout life in various mammalian species including humans [1,2,3,4]. Adult neurogenesis mainly occurs at two specific sites: the dentate gyrus (DG) of the hippocampus and the subventricular zone (SVZ) of the lateral ventricle (LV) [5,6]. Hippocampal neurogenesis, in particular, plays an important role in maintaining cognitive functions such as learning and memory [7]. In humans, aberrant hippocampal neurogenesis is associated with several diseases characterized by cognitive deficits, such as Alzheimer’s disease (AD), epilepsy, major depression disease, ischemic stroke, traumatic brain injury (TBI), and age-related decline in cognitive function [4,8,9,10].

Post-traumatic stress disorder (PTSD) is an anxiety disorder triggered by traumatic and threatening experience [11]. The disorder is characterized by symptoms of hyperarousal, social avoidance, and re-living the traumatic experience, reflecting memory abnormalities in these patients [12,13]. Hippocampal atrophy has been reported in PTSD patients, suggesting impaired hippocampal activity and neurogenesis. MRI studies have found reduced hippocampal volume in PTSD patient, and in functional memory task reduced hippocampal activation was detected by positron emission tomography (PET) imaging [14]. Increasing hippocampal neurogenesis has been proposed as a potential therapeutic strategy, validated in several animal studies [15,16,17]. It was also suggested that reduced hippocampal volume observed in PTSD patients is associated with systemic inflammatory changes [18]. Furthermore, Zhou et al. [19] have reported increased blood levels of inflammatory Th17 cells and their main pro-inflammatory cytokine derivative IL-17 in PTSD patients. Similar observations were reported in animal models for PTSD [20,21]. Th17 cells, the main producers of IL-17 cytokine, are a subset of the CD4^+^ T helper cells family, naturally involved in immune protection of barrier surfaces (mucosal tissues) against bacterial and fungal infections. Nevertheless, Th17 cells are considered as pro-inflammatory cells and are also involved in the pathophysiology of autoimmune diseases [22].

We previously discovered that a single administration of IL-17A in mice slightly improved spatial learning and altered neurogenesis by inhibiting proliferation of NSC, although increasing neurite growth and neuronal maturation. We suggested that IL-17 may act as a memory modulator, enabling the rapid acquisition of contextual cues while preventing their future modulation [23]. In the present study, we sought to explore the possible involvement of IL-17 in the effect of trauma exposure (inescapable electric foot shock) on hippocampal neurogenesis and its related behavior. We, therefore, attempted blocking IL-17A by administrating a neutralizing antibody to IL-17A or its upstream cytokine IL-23 prior to trauma exposure. Surprisingly, the anti-IL-17A antibody did not reduce serum levels of IL-17A but increased them (most likely due to protection from degradation). Thus, we were able to observe how sustained increase or decrease in IL-17A levels affected neurogenesis and behavior following exposure to trauma in mice.

## 2. Materials and Methods

### 2.1. Animals

**Figure 1:**
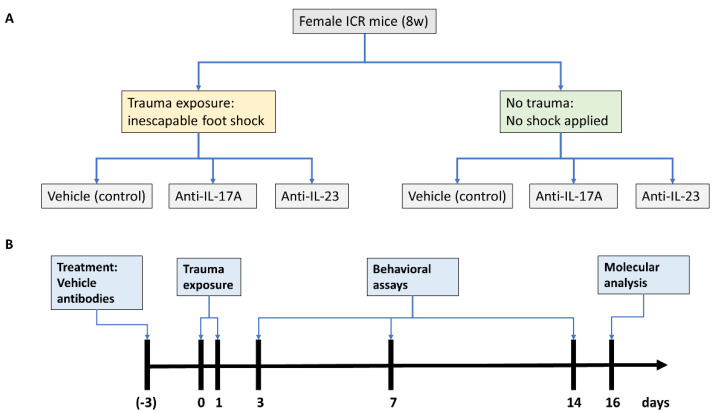
Experimental design. A scheme presenting all experimental groups (**A**) and experimental timeline (**B**).

All experimental procedures received ethical approval by Ariel University’s Animal Care and Use Committee and were performed according to the National Institutes of Health guidelines. Female ICR mice at the age of 8 weeks were purchased from Envigo (Jerusalem, Israel), and housed at a temperature of 22 °C under a 12:12 h dark:light cycle. Food (Teklad Global Diet from Envigo, Jerusalem, Israel) and water were provided ad libitum. We chose to perform our study in female mice since in humans, female subjects are more prone to develop PTSD [24] yet female animal studies are scarce [25,26]. Furthermore, since female rodents express a milder phenotype in response to inescapable electric foot shock [25], we expected the behavioral changes following cytokine manipulation to be more evident in females, in particularly exacerbated behavior. Three days prior to exposure to inescapable electric foot shock mice were injected intraperitoneally with either saline, 100 μg of anti-IL-17A neutralizing antibody (cat. No. BE0173. Bio X-cell, Lebanon, NH, USA), or 40 µg of anti-IL-23p19 neutralizing antibody (cat. No. 16-7232-85, eBiosciences, San Diego, CA, USA). The antibodies dose was adopted from previous studies [27,28]. We generated six experimental groups, three groups of which were exposed to trauma and treated with vehicle (control, 24 animals), anti-IL-17A (16 animals), and anti-IL-23 (12 animals). An additional three groups were not exposed to trauma but treated similarly with vehicle (control, 24 animals), anti-IL-17A (17 animals), and anti-IL-23 (12 animals) (Figure 1A).

### 2.2. Inescapable Electric Foot Shock

We adopted the inescapable electric foot shock model for PTSD as was previously described for ICR mice [29,30]. The electric shocks were applied by a PanLab (Barcelona, Spain) shocker apparatus (LE 100-26) at a current of 0.8 mA placed in a black, non-transparent, plastic arena. Female eight weeks old ICR mice were subjected to electric foot shock for two consecutive days (days 0 and 1), three days following antibody administration (Figure 1B). All mice underwent adaptation to the arena for 5 min. After 5 min, mice were subjected to 15 electric shocks at a duration of 10 s each, with 10 s of rest between the shocks. The total duration in the area was 5 min. The control mice subgroups were placed in the arena for a total of 10 min without applying electric shocks. Behavioral assays were conducted at days 3, 7, and 14 (Figure 1B).

### 2.3. Behavioral Assays

#### 2.3.1. Freezing Test

To assess the level of trauma related behavior, the mice were placed in the same arena where the electric foot shock took place, for 5 min, at consecutive days 3, 7, and 14. Freezing behavior was measured as the total inactivity duration recorded by a computerized video tracking system (EthoVision tracking system, Noldus; Wageningen, Netherlands).

#### 2.3.2. Elevated Plus Maze

Immediately at the end of the freezing test, the mouse was moved to the elevated plus maze (EPM). In the EPM, the level of general (non-contextual) anxiety and fear of the mouse was assessed. During this test, each mouse was placed for 5 min in a plus-shaped arena about half a meter from the floor with two open arms and two walled, closed arms at the length of 1 m. Mice were placed in the middle of the arena and allowed to move freely. The duration spent by the mouse in the opened arms relative to the total duration it spends in all arms was recorded by EthoVision video tracking system.

#### 2.3.3. Open Field Test

The open field test was applied to assess general locomotor activity and general anxiety. Mice were placed at the middle of a black arena measuring 40 × 40 × 40 cm for a period of 6 min. The total distance walked by the tested animal, and the time spent in the center, margin, and corners of the arena were recorded by EthoVision video tracking system.

#### 2.3.4. Social Interaction

Social interaction was assessed as the interaction duration of a tested mouse with a novel unfamiliar mouse. Prior to the beginning of the exam, the novel mouse is placed for 7 min in the arena for habituation. During the interaction session, any initiated contact from the trial mouse by nose or body (but not by tail) was defined as social interaction. The collective duration of interaction was recorded by observation.

### 2.4. IL-17A and IL-17F ELISA

IL-17A and IL-17F share structural and functional similarities. Both are expressed by Th17 cells and can both bind the IL-17RA receptor as homodimers. IL-17A and IL-17F can even form an AF heterodimer, although the IL-17A homodimer is considered the most potent activator of target cells [31]. We therefore analyzed the levels of both cytokine in serum samples. Interleukine-17 Modulates Neurogenesis and Behavio behavioral assay mice were anesthetized with a ketamine (150 mg/kg) and xylazine (10 mg/kg) mixture, and approximately 800 μL of blood was collected directly from the heart, incubated for 30 min at room temperature (RT) in plastic tube serum, and was harvested following blood coagulation. Fifty microliters of serum were then assessed for the levels of IL-17A and IL-17F by ELISA. For assessing antibody bound IL-17A complexes, 50 μL of diluted serum (diluted by ELISA buffer) was filtered through a 100KDa filter-membrane (Vivaspin500, Sartorius, Goettingen, Germany) according to the manufacturer’s protocol. Following filtration, the membrane bound proteins were resuspended with 50 μL of the dilution buffer, thus, acquiring the > 100 KDa fraction that was assayed by ELISA as well. For analyzing in vitro complexing, 20 ng of recombinant murine IL-17A (PeproTech, Cranbury, NJ, USA, cat. No. 210-17) was mixed and equilibrated for 10 min with anti-IL-17A antibody (1:1 ratio in 0.1 mL PBS), then filtered through 100 KDa filter-membrane. Filtrate was analyzed for IL-17A in ELISA. For analyzing the levels of serum IL-17A and IL-17F, ELISA kits for the detection of IL-17A (cat. No. 199081 Abcam, Cambridge, UK) and IL-17F (cat. No. M17F0 R&D systems, Minneapolis, MN, USA) were applied. All reagents were supplied from the manufacturer. In brief, for the detection of IL-17A, 50 µL of undiluted serum was applied in each well and 50 µL of Antibody Cocktail were added. The plate was incubated in RT for 1 h over a shaker. At the end of the incubation, the wells were washed with 1× Wash Buffer RT. 100 µL of TBM Substrate was added to each well and incubated in RT covered for 10 min. At the end, 100 µL of stop solution was added, optical absorbance was measured at 450 nm using TECAN (Männedorf, Switzerland) reader (infinite F200), and concentrations (pg/mL) were calculated according to prepared standard curve. For the quantification of IL-17F, 50 µL of Assay Diluent RD1-40 and 50 µL of the sample were added to each well and the plate was incubated for 2 h over a shaker at RT. The fluid was aspirated and washed four times with Wash Buffer. One hundred µL of Mouse/Rat IL-17F Conjugate was added to each well and incubated for 2 h over a shaker at RT. The wells were aspirated and washed once again. One hundred µL of Substrate Solution was added to each well and the plate was incubated for 30 min over a shaker, covered from light, at RT. Lastly, 100 µL of Stop Solution was added to each well, optical absorbance was measured at 450 nm using TECAN (Männedorf, Switzerland) reader (infinite F200), and concentrations (pg/mL) were calculated according to prepared standard curve.

### 2.5. Immunohistochemistry Staining

Immunohistochemistry (IHC) staining was performed to assess hippocampal neurogenesis. Following behavioral assays, mice were sacrificed by intracardial perfusion with saline followed by 4% paraformaldehyde (PFA) (cat. No. 47608-1L-F Sigma-Aldrich, Rehovot, Israel). Following brain removal, post fixation overnight, and equilibration in phosphate buffered 30% sucrose solution was commenced. Preparation of frozen brain tissue sections (20 μm) was conducted using MEV Slee Semi-Automatic Cryostat (SLEE medical GmbH, Nieder-Olm, Germany). Sections were stained for neuronal differentiation marker doublecortin (DCX) (cat. No. ab18723 Abcam, Cambridge, UK) and a proliferation marker Ki-67 (cat. No. ab15580 Abcam, Cambridge, UK) using an immunohistochemistry kit according to the manufacturer’s protocol (cat. No. MP-7451 Vector laboratories, Burlingame, CA, USA). Briefly, sections were fixed again with 4% PFA for 10 min followed by 10 min incubation with 3% H_2_O_2_. Membrane permeabilization was achieved by using 2% Triton X-100 for 5 min, blocking was then followed by incubation with a blocking solution (provided by manufacturer) for 45 min. Blocking was followed immediately by primary antibody incubation using rabbit polyclonal anti-DCX (diluted 1:500 in PBS) or primary rabbit polyclonal anti-Ki-67 (diluted 1:500 in PBS) overnight at 4 °C. On the following day, sections were incubated with horseradish peroxidase (HRP) one-step polymer conjugated secondary antibody for 30 min at room temperature. Following secondary antibody, the sections were incubated for 10 min with 3,3’ Diaminobenzidine tetrahydrochloride (DAB) buffer with DAB chromogen. Sections were washed three times with PBS for 5 min, between incubations. For the quantification of DCX and Ki-67 positive cells and nucleuses in the granular cell layer and subgranular zone, respectively, were counted on at least eight representing slides for each mouse and an average was calculated. Micrographs were acquired using an OLYMPUS BX53 microscope (Olympus, Tokyo, Japan) equipped with OLYMPUS camera U-TV0.5XC-3 with OLYMPUS CellSens imaging software.

### 2.6. Real-Time PCR Array for Neurogenesis-Related Genes

Total RNA was extracted from dissected hippocampi of CO_2_ sacrificed mice using an RNA Purification kit (cat. No. 47700, Norgen Biotech. Corp., Thorold, ON, Canada) according to the manufacturer’s protocol. RNA concentrations and quality were determined by absorbance at 260 nm, measured using the NanoDrop 2000 (Thermo Fisher scientific, Waltham, MA USA). Complementary DNA (cDNA) was transcribed from 1.5 µg RNA using the GoScript™ Reverse Transcription System (cat. No. A5003, Promega, Madison, WI, USA) and was subjected to real-time PCR analysis using the AriaMx 96 RT-PCR System (Agilent technologies Inc., Santa Clara, CA, USA) real-time PCR machine. Real-time PCR analysis was conducted for the expression of the following mouse genes: *Slit2, Hes1, Hey1, Ache, Dll1, Chrm-2*, *Il-17ra,* and hypoxanthine-guanine phosphoribosyl transferase (*Hprt*) as a reference gene. Samples were run in triplicates. Primers were purchased from Sigma-Aldrich (Rehovot, Israel) and are listed in Table 1. PCR amplification was performed using LightCycler SYBR Green I Master (cat No. 04887352001, Roche, Basel, Switzerland) under the following conditions: 5 min hot start at 95°C, 5 s denaturation at 95 °C, followed by 30 s annealing at 58 °C and then followed by 30 s elongation at 72 °C for a total of 40 cycles. PCR product specificity was confirmed using melting curve analysis. Relative gene expression was calculated as 2^(−∆ΔCT)^ [32].

### 2.7. Statistical Analysis

All data in the bar graphs is presented as mean with bars representing standard error. Statistical significance between two groups was calculated using the student’s *t*-test. Multiple comparisons between more than two groups were evaluated for significance using two-way analysis of variance (ANOVA) followed by Tukey’s and Sidak’s post-hoc test. Correlations were calculated using the Pearson correlation test.

## 3. Results

### 3.1. Long Term Changes in Serum Levels of IL-17A and IL-17F Following Exposure to Anti-IL-17A and Anti-IL-23

**Figure 2:**
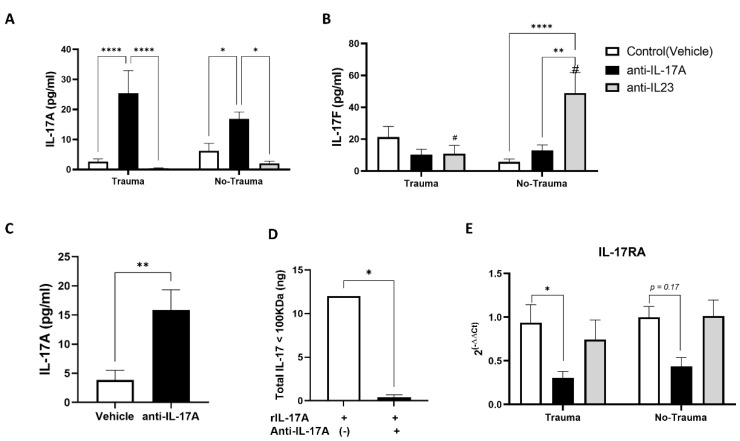
Antibody treatment affects IL-17 serum levels. Serum collected from mice at the end of the experiments (2.5 weeks post-trauma exposure) was analyzed for the protein levels of IL-17A (**A**) and IL-17F (**B**) using ELISA assay. Subgroups included pre-treatment with anti-IL17A antibodies (anti-IL17A), anti-IL-23 antibody (anti-IL-23), and vehicle injected animals (Control). (**A)** A graph presenting IL-17A serum levels detected by ELISA (anti-IL-17A, *n* = 12. anti-IL-23, *n* = 9, Control, *n* = 22). (**B**) IL-17F detection of the same sub-treatment groups as described in (**A**) (Trauma: anti-IL-17A, *n* = 9. anti-IL-23, *n* = 5, Control, *n* = 18. No trauma: anti-IL-17A, *n* = 11. anti-IL-23, *n* = 8, Control, *n* = 16). (**C**) Serum from control (vehicle treated animals) and anti-IL-17A treated animals was size filtered with 100 KDa cut-off membrane filter. The serum fraction containing protein complexes > 100 KDa was assayed for IL-17A levels using ELISA. The graph depicts the resulted concentrations. (anti-IL-17A, *n* = 11, Vehicle *n* = 9). (**D**) A graph presenting the total amount of recombinant murine IL-17A that was filtered by a 100 KDa membrane filter with or without pre-incubation with anti-IL17A antibody (1:1 ratio). A total amount of 20 ng rIL-17A was filtered, and filtrate was analyzed by IL-17A ELISA with the total amount of protein calculated (*n* = 2). (**E**) A graph presenting the relative gene expression for IL-17 receptor A (IL-17RA) in the hippocampus of animals from all treatment groups sacrificed 2.5 weeks post-trauma exposure, as detected by real-time PCR on hippocampal RNA. (Trauma: anti-IL-17A, *n* = 5. anti-IL-23, *n* = 5, Control, *n* = 12. No trauma: anti-IL-17A, *n* = 7. anti-IL-23, *n* = 6, Control, *n* = 11). All data in the graphs is presented as mean ± SE. * *p* < 0.05, ** *p* < 0.01, **** *p* < 0.0001. Paired symbols represent statistical significance between the two corresponding groups: # *p* < 0.05.

Female ICR mice at the age of 2 months were injected with either anti-IL-17A (100 μg) or anti-IL-23 (40 μg) and controls were injected with a vehicle three days prior to exposure to inescapable electric foot shock for two consecutive days. Two weeks later following behavioral assessment, serum was collected from the animals and serum levels of IL-17A and IL-17F were determined by ELISA (Figure 2A,B). We noticed that, surprisingly, administration of the anti-IL-17A neutralizing antibody increased the levels of IL-17A in the serum compared with anti-IL-23 and vehicle (control) treatments, regardless of trauma exposure (Figure 2A). On the other hand, anti-IL-23 treatment significantly increased IL-17F levels in mice not exposed to trauma (Figure 2B). We postulated that the IL-17A cytokine-antibody complex may protect the cytokine from degradation and form a reservoir of the cytokine in the serum. To confirm our hypothesis, we filtered the serum samples using a 100 KDa filtering membrane and assessed IL-17A in the >100 KDa fraction using ELISA (Figure 2C). In vitro testing showed that the cytokine-antibody complex is well retained in the >100 KDa fraction following membrane filtration and its filtration was prevented (Figure 2D). As we postulated, the levels of IL-17A in serum samples obtained from anti-iL-17A treated animals were significantly increased in the >100 KDa compared with control animals (Figure 2C). To assess whether high levels of IL-17A in animals treated with anti-IL-17A were biologically available and active in the brain, we analyzed the gene expression of the IL-17RA receptor in the whole hippocampus. Indeed, IL-17RA expression was down-regulated in anti-IL-17A treated animals exposed to trauma (Figure 2E). We thus refer to the anti-IL-17A subgroups as representing high serum levels of IL-17A.

### 3.2. Trauma Exposure and Antibody Treatment Altered Hippocampal Neurogenesis

**Figure 3:**
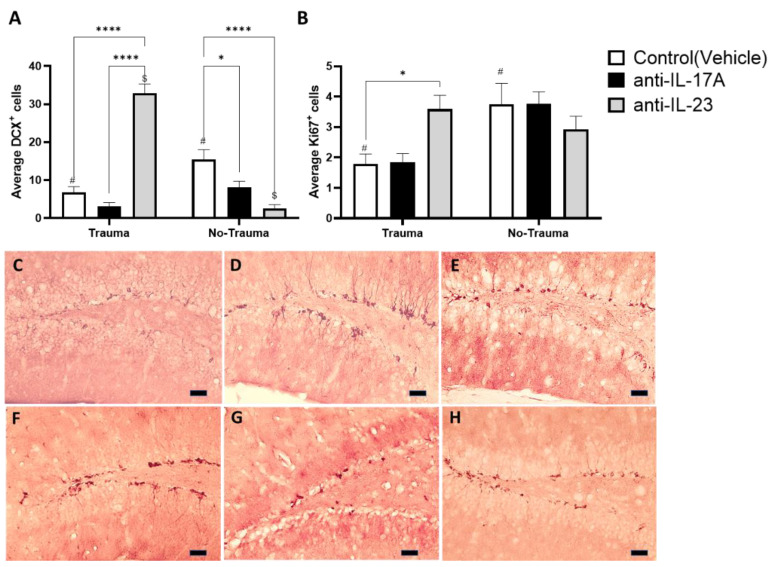
Exposure to trauma and antibody treatment affect hippocampal neurogenesis. Hippocampal neurogenesis was assessed by immunohistochemistry for proliferating neuro-progenitors (Ki67) and early differentiating neurons (DCX). (**A**) A graph presenting the average number of positive Ki67 cells in the sub-granular zone per hippocampal slide. (Trauma: anti-IL-17A, *n* = 5. anti-IL-23, *n* = 5, Control, *n* = 10. No trauma: anti-IL-17A, *n* = 3. anti-IL-23, *n* = 5, Control, *n* = 12). (**B**) A graph presenting the average number of positive DCX cells in the granular cell layer per hippocampal slide. Representing micrographs of DCX- stained hippocampi from trauma exposed mice treated with anti-IL-17A (**C**), anti-IL-23 (**D**), control (vehicle) (**E**) and control (no trauma) mice treated anti-IL-17A (**F**), anti-IL-23 (**G**) and control (vehicle) (**H**). All data in the graphs is presented as mean ± SE. * *p* < 0.05, **** *p* < 0.0001. Paired symbols represent statistical significance between the two corresponding groups: $ *p* < 0.05, # *p* < 0.05. Scale bars represent 20 μm.

Animals were sacrificed two weeks following exposure to trauma and behavioral tests. Analysis was performed on all treatment groups (Vehicle, anti-IL-17A, anti-IL-23) with and without exposure to trauma (i.e., electric foot shock). Brains were removed and assessed for hippocampal neurogenesis. The assessment of neurogenesis in the adult-hippocampus was performed by immunohistochemistry (IHC) staining, targeting Doublecortin (DCX), which is expressed in newly formed neurons in the granular cell layer (GCL), and Ki67, a marker for cell proliferation, in early progenitor cells in the sub granular zone (SGZ) of the dentate gyrus (DG). Ki67 was selected as a marker for proliferation instead of BrdU to avoid additional unwanted stress inflicted by BrdU injections. Trauma exposure was able to significantly reduce the number of DCX^+^ cells in the DG of vehicle treated animals (Figure 3A,E,H). Similarly, treatment with anti-IL-17A and anti-IL-23 mimicked the effect of trauma in animals not exposed to trauma (Figure 3A,F,G). However, an opposite effect was observed in trauma exposed animals that were treated with anti-IL-23 (Figure 3A,D). Treatment with anti-IL-23 significantly increased the number of DCX^+^ cells compared with its control group (no-trauma) and compared with other treatment groups (i.e., vehicle and anti-IL-17A). Trauma exposure also significantly reduced Ki67^+^ cells in the SGZ of vehicle treated mice and was close to significantly reduced (*p* = 0.06) in anti-IL-17A treated animals (Figure 3B). However, no reduction was observed in anti-IL-23 treated animals following exposure to trauma (Figure 3B).

### 3.3. Trauma Exposure and Antibody Treatment Altered the Expression of Neurogenesis-Related Genes in the Hippocampus

**Figure 4:**
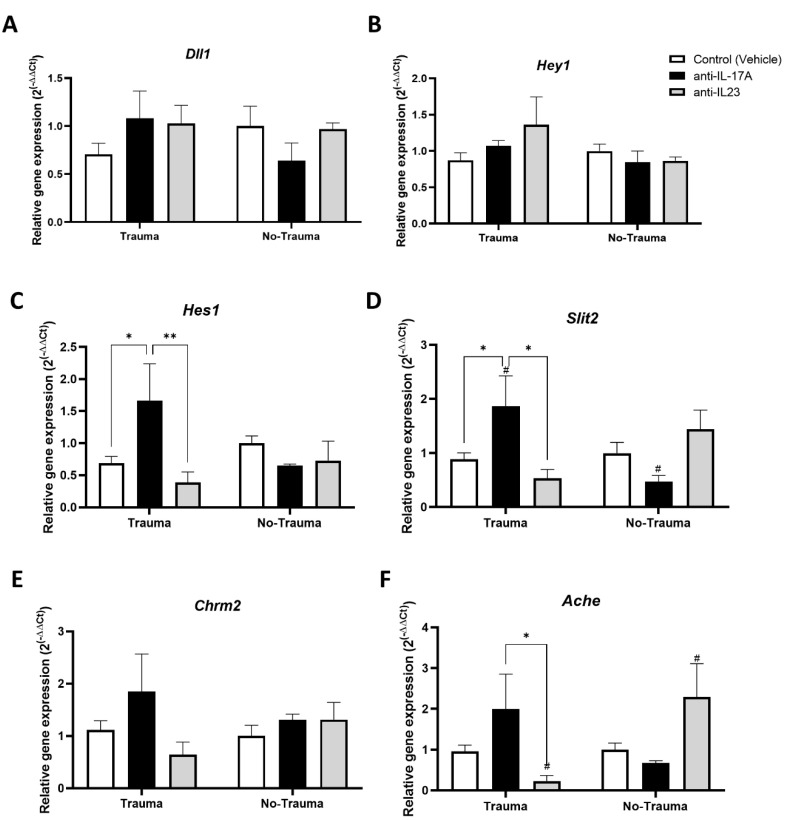
Exposure to trauma and antibody treatment affect hippocampal gene expression. Relative gene expression (2^−∆ΔCT^) detected by real time PCR performed on hippocampal RNA obtained from mice 2.5 weeks following exposure to trauma, for the following genes: (**A**) *Dll1* (Trauma: anti-IL-17A, *n* = 5. anti-IL-23, *n* = 5, Control, *n* = 8. No trauma: anti-IL-17A, *n* = 4. anti-IL-23, *n* = 6, Control, *n* = 12). (**B**) *Hey1* (Trauma: anti-IL-17A, *n* = 5. anti-IL-23, *n* = 6, Control, *n* = 9. No trauma: anti-IL-17A, *n* = 4. anti-IL-23, *n* = 6, Control, *n* = 12). (**C**) *Hes1* (Trauma: anti-IL-17A, *n* = 6. anti-IL-23, *n* = 5, Control, *n* = 9. No trauma: anti-IL-17A, *n* = 4. anti-IL-23, *n* = 6, Control, *n* = 11). (**D**) *Slit2* (Trauma: anti-IL-17A, *n* = 6. anti-IL-23, *n* =5, Control, *n* = 9. No trauma: anti-IL-17A, *n* = 4. anti-IL-23, *n* = 6, Control, *n* = 12). (**E**) *Chrm2* (Trauma: anti-IL-17A, *n* = 7. anti-IL-23, *n* = 6, Control, *n* = 9. No trauma: anti-IL-17A, *n* = 2. anti-IL-23, *n* = 5, Control, *n* = 12) and (**F**) *Ache* (Trauma: anti-IL-17A, *n* = 7. anti-IL-23, *n* = 6, Control, *n* = 11. No trauma: anti-IL-17A, *n* = 4. anti-IL-23, *n* = 6, Control, *n* = 12). All data in the graphs is presented as mean ± SE. * *p* < 0.05, ** *p* < 0.01. Paired symbols represent statistical significance between the two corresponding groups: # *p* < 0.05.

For assessing the expression of neurogenesis related genes, real-time PCR was performed on RNA extracted from the hippocampus. The NOTCH signaling pathway is an important regulator of neural stem cells proliferation, quiescence, and activity [33]. Since previous studies reported that hippocampal expression of *Notch* related genes and in particularly *Hes1* is regulated by IL-17 [23,34], we first analyzed the expression of NOTCH pathway related genes (Figure 4A–C). While the notch ligand *Dll1* and its downstream effector *Hey1* did not show any differences in expression (Figure 4A,B), *Hes1* was significantly increased in trauma exposed animals that were treated with anti-IL-17A (Figure 4C).

Our previous observation suggested that IL-17A can promote neuronal maturation and neurite growth [23]. We therefore sought to analyze the expression of genes associated with neurite guidance and cholinergic synapses, the main modulators of hippocampal plasticity and memory [35]. Interestingly, the neurite repellent *Slit2* was significantly increased following exposure to trauma in the anti-IL-17A treated subgroup (Figure 4D). Regarding cholinergic synapses, we did not find any effect of trauma exposure or treatment on the expression of the acetylcholine receptor M2 (*Chrm2*) (Figure 4E). However, a significant increase in acetylcholine esterase (*Ache*) expression was observed in anti-IL-17A subgroups following exposure to trauma, while decreased expression was observed in the anti-IL-23 subgroups (Figure 4F).

**Figure 5:**
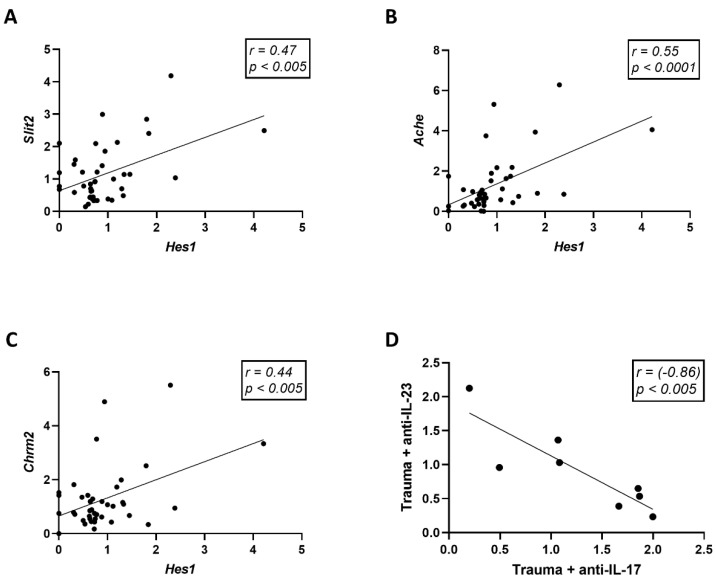
Gene expression correlations. Linear regression graphs depicting the correlation between *Hes1* gene expression and *Slit2* (**A**), *Ache* (**B**), and *Chrm2* (**C**) gene expression. (**D**) Changes relative to the control group (vehicle, no trauma) for the tested genes and immunohistochemistry (Ki67 and DCX) were plotted for anti-IL-17A and anti-IL-23 treatment groups in trauma exposed animals. Each dot represents a single neurogenesis parameter plotted for the change inflicted by anti-IL-17A treatment against the change inflicted by anti-IL-23 treatment. Correlations were calculated using Pearson test.

The expression of *Hes1* was found to be positively correlated with *Slit2* (Pearson *r* = 0.47, *p* < 0.005, *n* = 42), *Ache* (Pearson *r* = 0.55, *p* < 0.0001, *n* = 42), and *Chrm2* (Pearson *r* = 0.44, *p* < 0.005, *n* = 40) (Figure 5A–C). Interestingly, the overall changes in neurogenesis parameters inflicted by anti-IL-17A and anti-IL-23 treatments were antagonistic and inversely correlated in trauma-exposed animals (Pearson *r* = −0.86, *p* < 0.005, *n* = 8) (Figure 5D).

### 3.4. Trauma Exposure and Antibody Treatment Does Not Affect General Locomotion and Anxiety

**Figure 6:**
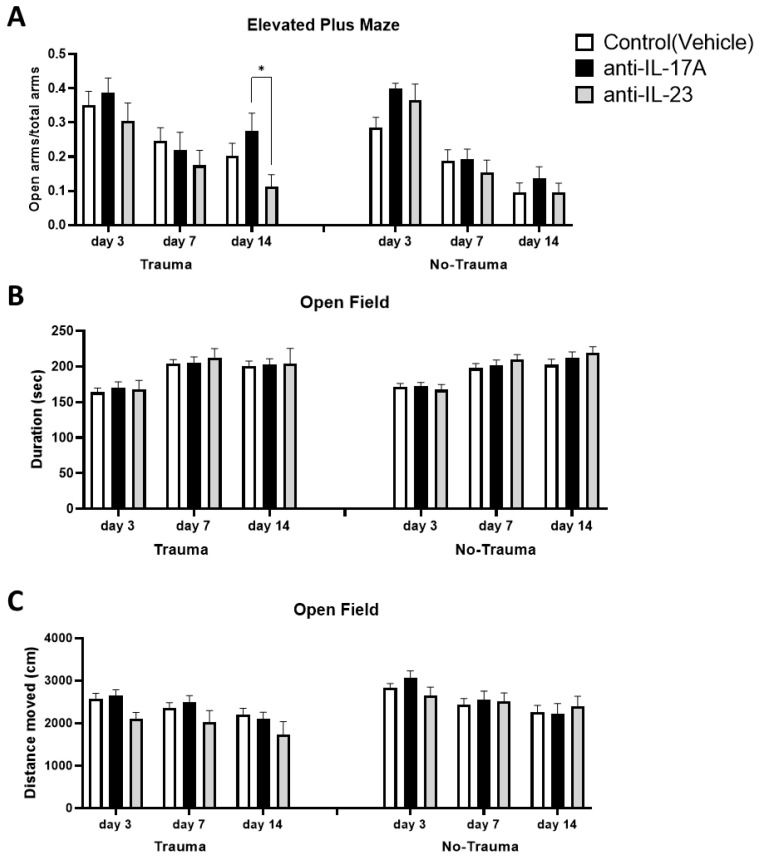
Exposure to trauma and antibody treatment do not affect general anxiety and locomotor activity. At days 3, 7, and 14 following the exposure to trauma, mice were subjected to elevated plus-maze and open field assays. General anxiety was measured in the elevated plus-maze assay where the preference of the tested mice for the open arms indicates for lower anxiety. (**A**) A graph presenting the ration of open arms duration to total arms duration of the various sub-groups. Similarly, the preference of the tested mice for the corners of the open field arena indicates for increased anxiety. (**B**) A graph presenting the duration the mice spent in the corners of the open field arena. (**C**) A graph presenting the total distance the mice travelled in the open field arena as an indication for locomotor activity. (Trauma: anti-IL-17A *n* = 16, anti-IL-23 *n* = 12, Control *n* = 24. No trauma: anti-IL-17A *n* = 17, anti-IL-23 *n* = 12, Control *n* = 24). All data in the graphs is presented as mean ± SE. * *p* < 0.05.

Female ICR mice at the age of 2 months were injected with either anti-IL-17A (100 μg) or anti-IL-23 (40 μg) and controls were injected with the vehicle three days prior to exposure to inescapable electric foot shock for two consecutive days (days 0 and 1). As a control for trauma, the similar treatment groups were subjected to the trauma arena but without applying electric shock. Open field and elevated plus-maze paradigms were applied at days 3, 7, and 14 following exposure (Figure 6). In the EPM test measuring general anxiety, no differences were observed between the subgroups regardless of trauma exposure, albeit a small difference between anti-IL-17A and anti-IL-23 14 days following exposure to trauma was noted (Figure 6A). Similarly, in the open field paradigm, no differences between the subgroups were observed in corner durations or general locomotion and no effect of trauma exposure could be seen either (Figure 6B,C).

### 3.5. Trauma Exposure Increased Trauma Related Anxiety

**Figure 7:**
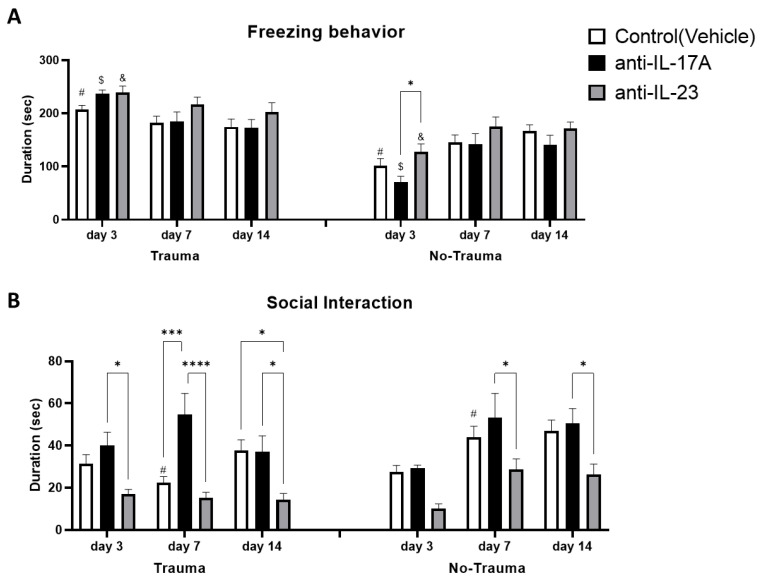
Exposure to trauma and antibody treatment affect trauma related behavior. At days 3, 7 and 14 following the exposure to trauma, mice were placed in the same arena in which they were exposed to the electric shock trauma for 5 min. Freezing behavior was measured as the duration of total inactivity by Ethovision^TM^ tracking system. (**A**) A graph presenting inactivity duration in the different sub-groups (Trauma: anti-IL-17A *n* = 15, anti-IL-23 *n* = 13, Control *n* = 24. No trauma: anti-IL-17-A *n* = 17, anti-IL-23 *n* = 14, Control *n* = 24). Social behavior was assessed as the duration of interaction between the tested mice and a novel unfamiliar mouse for 5 min, 3, 7- and 14-days following the exposure to trauma. (**B**) A graph presenting interaction duration in the different sub-groups (Trauma: anti-IL-17A *n* = 16, anti-IL-23 *n* = 12, Control *n* = 24. No trauma: anti-IL-17-A *n* = 17, anti-IL-23 *n* = 12, Control *n* = 24). All data in the graphs is presented as mean ± SE. * *p* < 0.05, *** *p* < 0.0005, **** *p* < 0.0001. Paired symbols represent statistical significance between the two corresponding groups: $ *p* < 0.05, & *p* < 0.05, # *p* < 0.05.

Trauma related anxiety was assessed as the freezing duration of mice when placed in the same arena where previous exposure to trauma was commenced, on days 3, 7, and 14. Trauma significantly increased freezing behavior in all sub-treatment groups early (day 3), following exposure to trauma but was gradually attenuated on days 7 and 14 following initial exposure. No significant differences were observed on day 3 between treatment groups exposed to trauma (Figure 7A).

### 3.6. Treatment with Anti-IL-17A Prevented Trauma-Induced Social Behavior Deficits While Anti-IL-23 Exacerbated Social Deficits

**Figure 8:**
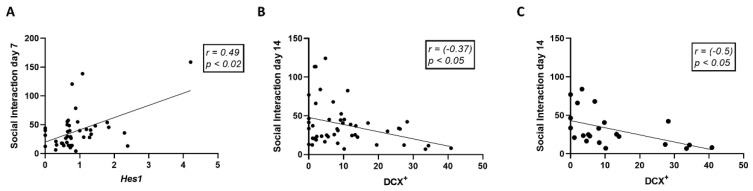
Social behavior correlates with hippocampal neurogenesis. Linear regression graphs depicting the correlation between *Hes1* gene expression and social interaction duration at day 7 (**A**). Correlation between the number of DCX^+^ cells in the dentate gyrus and social interaction duration at day 14 for all experimental groups (**B**) and trauma-exposed groups only (**C**). Correlations were calculated using Pearson test.

To assess social behavior impairment following trauma, we assayed mice in a simple social interaction paradigm, 3, 7, and 14 days following initial trauma exposure. At day 7, a significant reduction in social interaction duration was induced by trauma exposure in the vehicle subgroup, which was prevented in the anti-IL-17A treated subgroup (Figure 7B). On day 7 following trauma exposure, mice treated with anti-IL-17A exhibited increased social interaction compared with vehicle and anti-IL-23 subgroups. Mice treated with anti-IL-23 maintained low social interaction duration also on day 14, significantly lower than vehicle and anti-IL-17A subgroups. Interestingly, significant differences in social interaction between anti-IL-17A and anti-IL-23 treatments were also noted on days 7 and 14 in subgroups not exposed to trauma (Figure 7B). Social interaction was found to be positively correlated with *Hes1* gene expression (Pearson *r* = 0.49, *p* < 0.02, *n* = 42) on day 7 (Figure 8A). On the other hand, social interaction was negatively correlated with the average number of DCX^+^ cells on day 14 (Pearson *r* = −0.37, *p* < 0.05, *n* = 45) (Figure 8B). The negative correlation to DCX^+^ cells was even more prominent among the trauma exposed subgroups (Pearson *r* = -0.50, *p* < 0.05, *n* = 22) (Figure 8C).

## 4. Discussion

Owing to the key role of IL-17A in the pathophysiology of chronic inflammation and autoimmunity, therapeutic strategies targeting the pro-inflammatory cytokine were inevitably developed [31]. Among them, targeting of IL-17A with a neutralizing antibody was a promising approach [36]. In the present study we applied this approach to study the role of IL-17A in modulating neurogenesis and trauma-related behavior. Nevertheless, we found that anti-IL-17A antibody treatment increased the levels of serum IL-17A in mice (Figure 2A,B). Such a phenomenon was previously described for other cytokines and was attributed mainly to the protecting effect of the cytokine-antibody complex, resulting in increased half-life and availability of the cytokine [37,38]. Indeed, we found that IL-17A was present in the serum of anti-IL-17A treated animals in large complexes above 100 KDa (Figure 2C), which is well above its expected size of 35 kD [39]. Recently, a negative feedback of IL-17A/IL-17RA signaling was reported in Th17 cells as knockout of IL-17A in Th17 increased the IL-17RA expression [40]. Thus, the downregulation in the expression of the receptor IL-17RA in the hippocampus of mice treated with anti-IL-17A (Figure 2E) suggests that IL-17A complexes in these mice are bioactive rather than inactive.

In contrast to anti-IL-17A, anti-IL-23 administration resulted in reduced levels of IL-17A and F in mice exposed to trauma. Surprisingly, in control mice not exposed to trauma administration of anti-IL-23, IL-17A decreased but IL-17F increased (Figure 2A,B). Indeed, it was recently reported that downregulation of IL-23 in psoriatic lesions does not result in reduced IL-17 expression and suggests alternative regulatory pathways for IL-17 expression [41]. We therefore consider anti-IL-17A treatment as representing sustained increased levels of serum IL-17A, while anti-IL-23 represents lower levels in trauma-exposed mice.

Adult hippocampal neurogenesis is important for cognitive flexibility and pattern separation, and thus was suggested as a possible etiology for mood disorders including PTSD [30]. Studies in rodents previously demonstrated impaired adult hippocampal neurogenesis in PTSD models [15,16,17]. Similarly, we found that exposure to trauma reduced the proliferation of neural progenitors (ki67^+^) in the sub-granular zone and the number of newly formed neurons (DCX^+^) in the granular cell layer of the dentate gyrus (Figure 3). The involvement of IL-17A in this reduction is evident from the observations that anti-IL-17A treatment (i.e., increased IL-17A in the serum) mimicked the effect of trauma in control animals that were not exposed to trauma and significantly reduced the number of DCX^+^ cells (Figure 3A). Furthermore, anti-IL-23 treatment prevented the reduction in hippocampal neurogenesis in trauma exposed mice (Figure 3) and had an antagonistic effect on overall neurogenesis parameters than anti-IL-17A treatment (Figure 5D). The surprising reduction in DCX^+^ cells in control animals treated with anti-IL-23 can be similarly explained by the observed increase in IL-17F levels in these animals (Figure 2B).

Indeed, previous studies have demonstrated the inhibitory effect of IL-17 on neurogenesis, in particular, on neural progenitor proliferation in vivo and in vitro [23,34,42,43,44,45]. In the present study, we suggest that IL-17A’s effect can be partly explained by the upregulation of *Hes1* in trauma exposed animals treated with anti-IL-17A (Figure 4C). HES1, a downstream transcription factor of the NOTCH signaling pathway, is a known negative regulator of neurogenesis [46,47]. Nevertheless, we also observed an increase in the expression of genes associated with neuronal maturation such as the neurite repellent *Sli2* and Acetyl-choline esterase (*Ache*) in trauma exposed animals treated with anti-IL-17A (Figure 4). In addition, *Ache* expression was also increased in control animals not exposed to trauma that were treated with anti-IL-23, but presented high serum levels of IL-17F (Figure 2). We suggest that while early neurogenesis was inhibited by IL-17 (i.e., progenitor proliferation and early neuronal differentiation), maturation of already formed neurons was increased. Indeed, we previously proposed such a dual role of IL-17 on neurogenesis, as we observed it in vitro and in vivo in naïve animals that were treated with a single dose of IL-17A [23].

Exposure to electric foot-shock stress did not impair general anxiety and locomotor activity (Figure 6). It did, however, increase trauma-related freezing behavior and reduce social interaction 3 and 7 days following exposure to trauma, respectively (Figure 7). While both treatments with anti-IL-17A and anti-IL-23 did not affect freezing behavior, an opposite effect was observed on social interaction. Treatment with anti-IL-17A prevented social deficits on day 7 while anti-IL-23 exacerbated social deficits that were continued for 14 days following the exposure to trauma (Figure 7B). Interestingly, significant differences between the treatments were also noted in naïve mice that were not exposed to electric foot shock at all. We suggest that the increased levels of IL-17A present in the serum of anti-IL-17A exposed mice, as opposed to anti-IL-23 treated mice, are responsible for increased social interaction in anti-IL-17A treated animals. Indeed, we have previously reported on the possible positive effect of IL-17A on cognitive and affective behavior. We found that a single intravenous injection (8 μg) of IL-17A to naïve ICR mice resulted in improved spatial learning [23]. Furthermore, in a murine model for schizophrenia treated with mesenchymal stem cells, long term improved social behavior was achieved and was significantly correlated with increased hippocampal expression of IL-17 [48]. Indeed, it was recently reported that schizophrenia patients in remission following therapy had improved cognitive functions that were positively correlated with IL-17 serum levels [49]. Finally, a recent study demonstrated that local infusion of IL-17 to the somatosensory cortex of mice exhibiting impaired sociability due to maternal immune activation resulted in the reversal of social deficits [50].

Social interaction in our study was found to be inversely correlated with the number of newly formed DCX^+^ neurons in the dentate gyrus, and positively correlated with the NOTCH effector *Hes1* gene expression (Figure 8). While IL-17A expression is known to be regulated by NOTCH signaling, it was recently reported that it can activate NOTCH signaling as well [51]. Similarly, in the murine schizophrenia model treated with mesenchymal stem cells, long term improvement in social behavior was also positively correlated with the NOTCH ligand *Dll1* gene expression in the hippocampus [48]. Indeed, *Hes1* gene expression in mature neurons is important for maintaining behavior [52]. We propose that by promoting neuronal maturation and *Hes1* gene expression, IL-17A may prevent social deficits.

In conclusion, we suggest that IL-17 is involved in the deregulation of hippocampal neurogenesis induced by exposure to inescapable electric foot shock. Nevertheless, it may be crucial for the preservation of social behavior. However, our study was limited to female mice and further study is required to validate IL-17’s role in males as well. Future studies should also evaluate the role of peripheral immune cells as mediators of IL-17’s effect on neurogenesis and behavior. Finally, we propose that studying IL-17 signaling may be applied in humans for developing therapeutic strategies for treating social deficits in PTSD patients and other anxiety-related disorders.

## Figures and Tables

**Table 1:** Gene primer list.

Gene	Primer Sequence	
	Forward	Reverse
*Hprt*	TGTTGTTGGATATGCCCTTG	TTGCGCTCATCTTAGGCTTT
*Hes1*	CCAGCCAGTGTCAACACGA	AATGCCGGGAGCTATCTTTCT
*Chrm2*	CTGAAGGTGGCGGTTGACTT	TGGTTTGGCTATTACCAGTCCT
*Dll1*	TCATCACACCCTGGCAGACAGAT	ACGGAGAAGGTTGCTCTGTGTC
*Ache*	GAAGGCCGAGTTCCAC	GGCTCGGTCGTATTATATCC
*Hey1*	CCAGCCAGTGTCAACACGA	AATGCCGGGAGCTATCTTTCT
*Slit2*	AGGGAAGATGAGTGGCATTG	GTGCCTGAGACCAGCAAAAT
*Il-17ra*	GGACTGTGTGAACCGCTCTC	CCTGTGAAGTCAGTGGGAGG

## Data Availability

Not applicable.
